# Partial Qualitative Analysis of the Tomato

**Published:** 1851-05

**Authors:** Jno. T. Plummer

**Affiliations:** Richmond, Ia.


ARTICLE IX.

Partial Qualitative Analysis of the Tomato, (*Lycopersicum Esculentum—Solanum Lycopersicum.*)—By Jno. T. Plummer, M.D., of Richmond, Ia.

I have long wondered why the acid of a fruit so extensively used as the Tomato, should not have heretofore been determined. My earliest supposition was that the character of the acid had been ascertained, but that the course of my reading had not brought the analysis into my view. But years have passed, and I have not yet met with the slightest allusion to the quality of the acid, until to-day, in turning over the pages of the Transactions of the American Medical Association, I perceive that Dr. Porcher reports that this "fruit contains a *peculiar* acid." I have italicised the word "peculiar," because it implies, that whoever attempted the analysis must have failed to determine the true character of the acid; for, so far from being *peculiar* to the Tomato, it is common to very many acid fruits.

It may be that the reporter did not wish to imply that a chemical analysis had been made; but that, selecting his adjective rather carelessly, he merely intended to signify that the fruit contained an acid—an agreeable acid, or an unknown acid. Be this as it may, it appears that the Association gave, on this occasion, no additional information on the subject. The fact that the hundreds of intelligent physicians who composed the Association, allowed the statement to pass without note or comment, is presumptive evidence that the character of the tomato acid was not known to them. And if not known to them, to whom was it likely to be known?

Assuming, then, that no examination of the acid in question has been made public, I proceed to give the result of my own researches into the subject.

Every attentive person must have perceived, that the agreeable flavor of the Tomato is due to the semi-transparent mass that occupies and often fills the seed cavities, and envelopes the seed. In this translucent pulp, the acid is to be found. The parenchymatous portion of the fruit does indeed contain acid enough to redden litmus, but not enough to be perceptible to the taste.

The yellow Tomato was the variety upon which I operated.

1. The glair of the ripened fruit was subjected to pressure in a clean muslin cloth, and the acid juice obtained was then boiled in a Berlin evaporating dish, to coagulate the albumen present. Of this there was a considerable quantity, but it was easily separable by heat—the acid present no doubt facilitating the process.

2. The liquor was then filtered through paper, limpid and colorless. Tested with litmus paper, it proved to be strongly acid. This, indeed, was obvious to the taste.

3. This acid liquor was neutralized with ammonia. Both this alkali and potash gave to the liquor a wine-red color, which was discharged by an addition of the tomato juice, or other acid.

4. To the neutralized liquor (3) was added chloride of lime. This dissipated the wine-red color, but produced no precipitate. Ebullition in a test-tube, however, for a few moments, yielded a white precipitate. This experiment indicated the absence of oxalic, malic, tartaric and paratartaric acids, and the presence of citric acid.

5. The white precipitate (4) was soluble in chloride of ammonium. This solution boiled, again yielded a white precipitate. This reaction with sal-ammoniac afforded another evidence of the absence of paratartaric (racemic) acid.

6. The ebullition of 4 was continued until no more precipitate fell. To the decanted liquor alcohol was added, but the liquid remained clear. This furnished additional evidence of the absence of malic acid.

7. The acid juice (2) was neutralized with lime water. No precipitate appeared. On boiling, flocculi were produced, and these were redissolved on cooling. This reaction indicates citric acid, to the exclusion of almost every other organic acid.

8. The acid juice (2) was treated with acetate of lead. A very copious, heavy, white precipitate instantly fell. This precipitate was readily soluble in citrate of ammonia; thus again denoting the presence of citric acid.

9. To the filtered, neutralized juice, was added sesquichloride of iron. The liquid assumed a yellowish-green color, and remained perfectly transparent. The absence of any reaction in this case excludes the idea of tannic, gallic, acetic and benzoic acids being present.

Thus, then, I determined the certain existence of citric acid in the tomato, and the absence of all other acids. Other reagents were employed besides those named ; but, as they all produced corroborative evidence of the presence of citric acid, to the exclusion of others, I have not thought it necessary to add their indications to the foregoing.

It now became an interesting question, whether the acid discovered was wholly free, or in combination with a base. To resolve this problem, I added to the acid juice (2) a solution of tartaric acid in excess, and strongly agitated the mixture. A granular precipitate was formed, characteristic of potash. Tartrate of lime would have re-dissolved in the excess of tartaric present, and would also have disappeared in sal-ammoniac solution, which did not occur with the present precipitate.

Citrate of Potash, then, with excess of Citric Acid, is the salt which gives to the Tomato its agreeable flavor.

George Dow (General History of Dichlamydeous Plants, in four ponderous volumes, London, 1831,) says the esculent tomato was cultivated as early as 1596. Can it be possible that so much time has elapsed, and this fruit has been so very generally relished in different nations, and yet no one has heretofore been prompted to examine into the cause of its palatableness?

I have some further observations to make on this plant, and especially on its medicinal properties; but they will, perhaps, be more appropriate on another occasion.—*Western Lancet*, Jan., 1851.

